# Perioperatives Management der trochantären Femurfraktur: Nüchternheitsregime, Flüssigkeitstherapie und multimodale Schmerzkonzepte als unterschätzte Stellschrauben

**DOI:** 10.1007/s00113-026-01732-9

**Published:** 2026-08-03

**Authors:** Björn-Christian Link, Pascal C. Haefeli, Manuela Rohner-Spengler, Bryan van de Wall, Frank J. P. Beeres

**Affiliations:** 1 Klinik für Orthopädie und Unfallchirurgie, Luzerner Kantonsspital, Spitalstrasse, 6000 Luzern 16, Schweiz; 2 Fakultät für Gesundheitswissenschaften und Medizin, Universität Luzern, Luzern, Schweiz

**Keywords:** Hüftfraktur, Fascia iliaca, „Pericapsular nerve“, Postoperatives Delir, „Enhanced recovery after surgery“, Hip fractures, Fascia iliaca, Pericapsular nerve, Postoperative delirium, Enhanced recovery after surgery

## Abstract

**Hintergrund:**

Die trochantäre Femurfraktur ist die häufigste hüftnahe Fraktur des älteren Menschen und mit einer hohen perioperativen Morbidität und Letalität assoziiert. Während chirurgische Versorgungstechnik, Antikoagulation und orthogeriatrische Strukturen viel diskutiert werden, bleiben die pflegerischen und organisatorischen Stellschrauben des perioperativen Pfades, namentlich Nüchternheit, Flüssigkeitstherapie und Schmerzmanagement, oft unterbelichtet.

**Fragestellung:**

Welche Evidenz besteht für eine liberalisierte präoperative Nüchternheit („sip till send“), für eine zielorientierte Volumentherapie und für den Einsatz peripherer Nervenblockaden mit und ohne Schmerzkatheter im Hinblick auf Schmerzkontrolle, postoperatives Delir und funktionelles Ergebnis?

**Material und Methoden:**

Selektives narratives Review der Literatur der letzten 10 Jahre einschließlich systematischer Reviews, randomisierter Studien und nationaler Empfehlungen.

**Schlussfolgerung:**

Klare Flüssigkeiten bis zur Abrufung in den Operationssaal sind sicher und reduzieren Durst, Hunger und Volumendepletion. Eine liberale intraoperative Volumengabe ist mit höheren Komplikationsraten vergesellschaftet, eine zielorientierte Volumentherapie mit balancierten Lösungen ist daher zu bevorzugen. Periphere Nervenblockaden reduzieren Schmerz, Opioidbedarf und in mehreren Metaanalysen die Inzidenz des postoperativen Delirs. Kontinuierliche Schmerzkatheter sind eine attraktive Option bei längerer präoperativer Wartezeit. Eine konsequente Bündelung dieser Maßnahmen im Sinne eines Enhanced-recovery-Konzepts ist mit verkürzter Verweildauer und besserer Erholung assoziiert.

## Abstract (en)

**Background:**

Trochanteric femoral fracture is the most frequent proximal femur fracture in older adults and is associated with substantial perioperative morbidity and mortality. While surgical technique, anticoagulation and orthogeriatric care are widely discussed, the nursing and organisational levers of the perioperative pathway, namely fasting, fluid therapy and pain management, often remain underappreciated.

**Objective:**

To review the evidence on a liberalised preoperative fasting policy (sip till send), goal-directed fluid therapy, and peripheral nerve blocks with and without continuous catheters with regard to pain control, postoperative delirium and functional outcome.

**Methods:**

Selective narrative review of literature published within the last decade, including systematic reviews, randomised trials and national recommendations.

**Conclusion:**

Clear fluids up to the call to theatre are safe and reduce thirst, hunger and volume depletion. Liberal intraoperative fluid administration is associated with higher complication rates; goal-directed therapy with balanced crystalloids is therefore preferable. Peripheral nerve blocks reduce pain, opioid consumption and the incidence of postoperative delirium. Continuous nerve catheters are an attractive option in case of prolonged preoperative waiting times. Bundling these measures within an enhanced-recovery framework is associated with shorter length of stay and improved recovery.

## Einleitung

Die pertrochantäre Femurfraktur (AO/OTA 31-A) ist die häufigste hüftnahe Fraktur des betagten Patienten und ein paradigmatischer Eingriff der modernen Alterstraumatologie. Die 30-Tage-Letalität liegt international zwischen 5 und 10 %, die Ein-Jahres-Letalität zwischen 20 und 30 % [1, 2]. Während sich die operative Versorgung mit zephalomedullären Marknägeln in den letzten 2 Dekaden weitgehend standardisiert hat und die Diskussion derzeit auf die Zementaugmentation [3], die frühe Versorgung unter direkten oralen Antikoagulanzien [4] und die Strukturen einer orthogeriatrischen Co-Therapie [5] fokussiert ist, geraten die pflegerischen, anästhesiologischen und organisatorischen Komponenten des perioperativen Pfades regelmäßig in den Hintergrund.

Gerade diese Komponenten, namentlich kurze Zeit zwischen Fraktur und operativer Versorgung, präoperative Nüchternheit, Volumentherapie und Schmerztherapie, prägen indes die Tage zwischen Spitaleintritt und gelungener Mobilisation entscheidend. Sie entscheiden über generelles Komplikationsrisiko, Aspirationsrisiko, perioperative Hypotonie, Akutschmerzlast, Opioidexposition und damit über das Risiko eines postoperativen Delirs [6, 7]. Die vorliegende Übersicht fasst die aktuelle Evidenz zu drei Themenfeldern zusammen, die in der täglichen Praxis häufig unterbelichtet bleiben: dem Konzept „*sip till send*“, der zielorientierten perioperativen Volumentherapie sowie den peripheren Nervenblockaden mit und ohne Schmerzkatheter. Verzichtet wird bewusst auf eine erneute Diskussion der Zementaugmentation, des Vorgehens unter direkten oralen Antikoagulantien (DOAK) und der orthogeriatrischen Strukturen, die in dieser Themenausgabe in eigenen Beiträgen behandelt werden.

## Der Patient zwischen Eintritt und Operation: Ein vulnerables Zeitfenster

Eine zeitnahe operative Versorgung innerhalb von 24 bis 48 h ist ein etablierter Qualitätsindikator [1, 8]. Auch bei einer Versorgung im empfohlenen Zeitfenster verbringt der Patient typischerweise 12 bis 36 h zwischen Triage, Diagnostik, anästhesiologischer Vorbereitung und Operationsbeginn. Diese Phase ist kein passiver Wartezustand, sondern ein aktiv zu gestaltendes Zeitfenster: Schmerz, Volumendepletion, Hunger, Schlafentzug und Immobilisation summieren sich zu einer kumulativen Stressbelastung, die das Auftreten eines Delirs begünstigt und die postoperative Erholung verzögert [6, 9].

Drei in der täglichen Routine getroffene Entscheidungen sind besonders folgenreich: Wann und wie wird Nahrung und Flüssigkeit karenziert? Wie wird der Volumenhaushalt aktiv gesteuert? Welche Schmerztherapie steht in welchem Zeitfenster zur Verfügung? Diese drei Achsen werden in den folgenden Abschnitten beleuchtet, im Anschluss synthetisiert in einem Bündel im Sinne von Enhanced Recovery After Surgery (ERAS®).

## Präoperative Nüchternheit: Vom dogmatischen „nil-by-mouth“ zum „sip till send“

### Wo wir herkommen

Die Verordnung einer kompletten Nahrungs- und Flüssigkeitskarenz ab Mitternacht ist eine der zählebigsten Routinen der perioperativen Medizin. Sie geht auf eine Zeit zurück, in der Anästhesietechnik, Atemwegssicherung und Antiemese deutlich weniger ausgereift waren. Die nationalen und internationalen Fachgesellschaften (American Society of Anesthesiologists [ASA], European Society of Anaesthesiology and Intensive Care [ESA/ESAIC], Association of Anaesthetists of Great Britain and Ireland [AAGBI]) empfehlen seit über 2 Jahrzehnten eine Karenz von 6 h für feste Nahrung und von 2 h für klare Flüssigkeiten [10, 11]. In der Realität werden gerade Patienten mit trochantärer Femurfraktur jedoch häufig 8, 12 oder mehr Stunden vollständig nüchtern gehalten [9, 11].

Die Folgen sind nicht trivial. Verlängerte Fastenzeiten führen bei alten, oft bereits präoperativ exsikkierten Patienten zu Hypovolämie und damit zu erhöhter perioperativer Hypotonie, Nierenfunktionsverschlechterung und Delir [9]. Hinzu kommen Durst, Hunger, Übelkeit, Kopfschmerz und ein subjektiv als entwürdigend empfundener Zustand, der die Adhärenz an die Behandlung schwächt [11].

### „Sip till send“: Das Konzept

Vor diesem Hintergrund hat sich, ausgehend von Schottland und Australien, das Konzept „sip till send“ (auch „drink until called“) etabliert [9, 11, 12]. Geeignete erwachsene Patienten dürfen bis zum Abruf in den Operationssaal kleine Mengen klarer Flüssigkeit (in der Regel bis zu 200 ml/h aus einem Standardbecher) trinken. Die feste Nahrungskarenz von 6 h bleibt unverändert [11, 12].

Das Konzept stützt sich auf zwei pathophysiologische Beobachtungen. Erstens ist Wasser ein neutrales Medium mit sehr kurzer Magenpassagezeit; auch bei Patienten mit Hüftfraktur, die ein verzögertes Magenentleerungsprofil aufweisen können, führt Sippen kleiner Mengen nicht zu einer relevanten Zunahme des Magenvolumens [11]. Zweitens ist das Aspirationsrisiko unter modernen Anästhesieverfahren mit Atemwegssicherung sehr gering, und es findet sich in der verfügbaren Evidenz kein Hinweis auf eine Erhöhung dieses Risikos durch „sip till send“ im Vergleich zur klassischen Karenz [9, 11, 12].

### Evidenzlage und Implementierung

Eine zunehmende Zahl an Beobachtungs- und Qualitätsverbesserungsstudien dokumentiert die Sicherheit und den Nutzen einer abgekürzten Flüssigkeitskarenz. In einem prospektiven Kohortenprojekt im Schottischen Hüftfraktur-Audit wurde die mediane Flüssigkeitskarenz nach Einführung von „sip till send“ von über 6 auf weniger als 2 h reduziert, ohne dass eine Zunahme von Aspirationsereignissen auftrat [9]. Eine multizentrische Untersuchung an chinesischen Standorten zur abgekürzten perioperativen Karenz bei elektiven Frakturen zeigte ebenfalls eine hohe Adhärenz und keine Zunahme von Reflux- oder Aspirationskomplikationen [13]. Auch eine Machbarkeitsstudie zur präoperativen Kohlenhydrat-Belastung bei älteren Patienten mit Hüftfraktur unterstrich die Sicherheit dieses Vorgehens [14].

Die Hürden liegen weniger in der Evidenz als in der Implementierung. „Sip till send“ erfordert eine multidisziplinäre Konsentierung zwischen Anästhesie, Orthopädie/Unfallchirurgie und Pflege, klare Algorithmen für Patienten mit verzögerter Magenentleerung (Diabetes mellitus mit autonomer Neuropathie, Behandlung mit GLP-1-Rezeptoragonisten, Ileussymptomatik) und eine konsequente Schulung des Personals einschließlich gut sichtbarer Bettkennzeichnung und einer hinterlegten elektronischen Verordnung [12, 15]. In jedem Fall bleibt die individuelle Risikoabwägung durch den Anästhesisten unbenommen.

Aus unserer Sicht sollte „sip till send“ heute der Default-Pfad für Patienten mit trochantärer bzw. koxaler Femurfraktur sein, abweichend nur bei dokumentierten anästhesiologischen Bedenken. Die alleinige Vermeidung iatrogen verlängerter Fastenzeiten ist eine der kostengünstigsten Maßnahmen mit dem größten Patient-reported-Benefit, die im perioperativen Pfad zur Verfügung steht.

## Perioperatives Flüssigkeitsmanagement: Zwischen Skylla und Charybdis

### Ausgangslage des betagten Patienten

Der typische Patient mit trochantärer Femurfraktur ist gebrechlich, multimorbid, häufig kardial oder renal eingeschränkt und nicht selten bereits bei Eintritt hypovoläm. Sturzgeschehen, präklinische Wartezeit, Schmerzen, eingeschränkte Trinkmenge und gegebenenfalls eine vorbestehende Diuretikatherapie summieren sich. Das Volumenmanagement bewegt sich daher zwischen zwei Extremen, deren Vermeidung beide gleichermaßen wichtig sind: Hypovolämie mit Hypotonie, Nierenfunktionsverschlechterung und Delirrisiko auf der einen Seite, iatrogene Hypervolämie mit Lungenödem, Hyponatriämie und kardialer Dekompensation auf der anderen Seite [16, 17].

### Liberale Volumengabe als Risikofaktor

Die wohl wichtigste rezente Arbeit zu dieser Frage stammt aus dem deutschsprachigen Raum: Pass et al. [16] analysierten in einer multizentrischen Kohorte die intraoperative Flüssigkeitsgabe bei geriatrischen Hüftfrakturpatienten und konnten zeigen, dass ein liberales Regime, definiert als intraoperative Gabe von > 1500 ml Kristalloid, mit signifikant höheren Raten an Transfusionsbedarf, Verlegung auf eine Überwachungseinheit und Gesamtkomplikationen einherging. Die Autoren plädieren überzeugend für ein restriktiveres, zielorientiertes Vorgehen.

### Ziele einer modernen Volumentherapie

In der Praxis bewährt hat sich ein Konzept, das die folgenden Eckpunkte umfasst: Erstens, eine frühzeitige Korrektur einer Eintrittsdehydratation bereits in der Notfallaufnahme mittels balancierter Vollelektrolytlösung (in der Regel 500–1000 ml in der ersten Stunde, je nach hämodynamischem Status). Wo immer möglich, ergänzt durch orale Flüssigkeitsaufnahme im Sinne von „sip till send“.

Zweitens, intraoperativ eine zielorientierte Volumentherapie unter klinischer und gegebenenfalls erweiterter hämodynamischer Steuerung (mittlerer arterieller Druck, Diurese, Laktat, gegebenenfalls Schlagvolumenvariation), mit dem Ziel der Normovolämie und nicht der Übersubstitution.

Drittens, eine restriktive Verwendung kolloidaler Lösungen, da der Nutzen gegenüber balancierten Kristalloiden in dieser Population nicht belegt ist und Risiken (insbesondere Nierenfunktionsverschlechterung) bestehen.

Viertens, eine konsequente Vermeidung von 0,9-prozentiger Kochsalzlösung als Grundlösung wegen des Risikos hyperchlorämischer Azidose, insbesondere bei der häufig vorbestehenden Niereninsuffizienz.

Postoperativ ist die zügige Reduktion der parenteralen Volumengabe zugunsten einer frühzeitigen oralen Flüssigkeits- und Nahrungsaufnahme ein Element jedes ERAS-Konzepts [17, 18]. Tägliche Volumenbilanzen, Gewichtsmonitoring und ein wachsamer Blick auf Hinweise einer Überwässerung gehören zur orthogeriatrischen Routine.

## Schmerzmanagement: Multimodalität statt Opioidmonotherapie

### Schmerz als prognostische Variable

Schmerz ist bei der trochantären Femurfraktur nicht bloß ein Komfort-, sondern ein Outcome-Parameter. Unkontrollierter Schmerz erhöht das Delirrisiko, behindert die frühzeitige Mobilisation und prolongiert den Krankenhausaufenthalt [6, 19]. Eine konsequente, frühe und mehrstufige Schmerztherapie ist daher ein zentraler Hebel im perioperativen Pfad.

Die alleinige systemische Opioidgabe stößt beim alten Patienten an enge Grenzen. Sie bringt Sedierung, Atemdepression, Übelkeit, Obstipation und, besonders relevant, eine signifikante Erhöhung des Delirrisikos mit sich [6, 20]. Gleichzeitig sind nichtsteroidale Antirheumatika in dieser Population häufig kontraindiziert (Niereninsuffizienz, kardiovaskuläre Komorbidität, gastrointestinale Risiken). Ein multimodales Konzept, das Paracetamol als Basismedikation, restriktive Opioidgabe und früh eingesetzte regionalanästhesiologische Verfahren kombiniert, ist heute Standard [6, 18, 19].

### Femoralisblock (FNB): Der historische Klassiker

Über mehr als 2 Jahrzehnte war der ultraschall- oder nervenstimulatorgesteuerte Femoralisblock („femoral nerve block“, FNB) die regionale Referenztechnik bei der hüftnahen Fraktur. Er deponiert das Lokalanästhetikum direkt am N. femoralis lateral der A. femoralis im Bereich der Inguinalfalte und ist technisch einfach, gut zu erlernen und mit niedriger Komplikationsrate durchführbar [21]. Mehrere randomisierte Studien und Metaanalysen belegen seine Wirksamkeit gegenüber rein systemischer Analgesie hinsichtlich Schmerzscore, Opioidbedarf und Erleichterung der Lagerung für die Spinalanästhesie [21, 22].

Im direkten Vergleich mit dem FICB (Fascia-iliaca-compartment-Block) zeigen aktuelle Metaanalysen weitgehend äquivalente Ergebnisse: Cui et al. ([32]; 15 RCT [„randomised controlled trial“], 1240 Patienten) fanden einen geringen Vorteil des FICB bezüglich VAS-Score (visuelle Analogskala) zur 4. postoperativen Stunde, ansonsten ohne klinisch relevante Unterschiede. Hong und Ma ([33]; 14 RCT, 1179 Patienten) berichten umgekehrt einen leichten Vorteil des FNB bezüglich Schmerzscore in Ruhe nach 24 h sowie geringerer Inzidenz von Übelkeit und Sedierung. Die Wahl zwischen FNB und FICB ist daher in erster Linie eine Frage der lokalen Expertise, nicht der Wirksamkeit.

Eine bedeutsame Limitation teilt der FNB mit dem klassischen FICB: Er bewirkt eine Schwächung des M. quadriceps femoris. Dies fällt beim Single-shot-Block in der Notfallphase kaum ins Gewicht, gewinnt aber beim kontinuierlichen Verfahren erhebliche Bedeutung (s. Abschnitt zu Schmerzkathetern).

### Fascia-iliaca-compartment-Block (FICB): Infrainguinal und suprainguinal

Der FICB ist die am besten untersuchte regionale Technik in der präoperativen Phase. Eine Cochrane-Übersicht und mehrere Metaanalysen belegen konsistent eine Reduktion des Schmerzscores in Bewegung, eine Verminderung des präoperativen Opioidbedarfs und eine Erleichterung der Lagerung für die Spinalanästhesie [21, 22]. Die Erfolgsrate ist hoch, das Komplikationsrisiko niedrig, und der Block kann nach kurzer Schulung auch in der Notfallaufnahme zuverlässig durchgeführt werden [22].

Inhaltlich ist zwischen dem klassischen infrainguinalen Zugang und der suprainguinalen Variante (SI-FICB) zu unterscheiden. Die suprainguinale Technik erlaubt eine zuverlässigere kraniale Ausbreitung des Lokalanästhetikums und damit eine vollständigere Erfassung der relevanten Nerven (N. femoralis, N. obturatorius, N. cutaneus femoris lateralis). Sie ist die heute bevorzugte Variante bei der trochantären Fraktur [23].

### PENG-Block: Motorisch sparende Alternative

Eine jüngere Entwicklung ist der Pericapsular-nerve-group-Block (PENG), erstmals 2018 beschrieben. Die Lokalanästhetikumdeposition in der muskulofaszialen Schicht zwischen M. psoas und Os pubis zielt selektiv auf die artikulären Äste zur Hüftgelenkkapsel und schont die motorische Funktion des N. femoralis weitgehend [24, 25]. Daraus ergibt sich der konzeptionelle Vorteil, dass der Patient früh und sicher mit Quadrizepskontrolle mobilisiert werden kann, was im Hinblick auf die postoperative Frührehabilitation von Bedeutung ist.

Die randomisierte Evidenz zwischen PENG und SI-FICB ist heterogen. Eine aktuelle systematische Übersicht und Metaanalyse aus dem Jahr 2025 [25] sowie randomisierte Vergleichsstudien [24, 26] sprechen dem PENG-Block einen Vorteil in der unmittelbaren Schmerzreduktion und in der Erleichterung der Lagerung für die Spinalanästhesie zu, bei vergleichbarer Sicherheit. Beide Techniken sind kompatibel mit einer therapeutischen Antikoagulation, sofern die Empfehlungen der Fachgesellschaften zu Punktion und Lokalanästhetikum-Mengen beachtet werden.

### Schmerzkatheter und kontinuierliche Blockaden

Während die Single-shot-Blockade in der Regel sechs bis 12 h Wirkung entfaltet, kann das Versorgungsfenster bei einer trochantären Fraktur deutlich länger sein, sei es durch verzögerte Operationsplanung, längere Wartezeiten unter DOAK-Pause oder ausgeprägte Beschwerden in den ersten 24 bis 48 postoperativen Stunden. Hier kommen kontinuierliche Verfahren ins Spiel: Prinzipiell als Femoraliskatheter (cFNB), als FICB-Katheter oder, zunehmend, als PENG-Katheter mit kontinuierlicher Infusion eines niedrig dosierten Lokalanästhetikums (typischerweise Ropivacain 0,1–0,2 %, 4–8 ml/h; [25, 27, 34]).

#### Femoraliskatheter (cFNB).

Der kontinuierliche Femoralisblock ist die historisch am längsten etablierte Kathetertechnik mit standardisierten Anlageschemata und gut beschriebenen Resultaten. Sein zentraler Schwachpunkt für die Hüftfrakturpopulation liegt jedoch in der ausgeprägten motorischen Blockade: Auch unter niedrig dosiertem Ropivacain 0,1 % bei 4 ml/h induziert der cFNB eine Quadrizepsschwäche von 70–80 % des Ausgangswerts [34]. Beim 80-jährigen Patienten, der am Operationstag oder spätestens am ersten postoperativen Tag mobilisiert werden soll, übersetzt sich diese motorische Blockade unmittelbar in ein erhöhtes Sturzrisiko und damit in den potentiellen Verlust eines der wichtigsten Outcome-Vorteile des Gesamtkonzeptes [21]. Wo ein cFNB eingesetzt wird, sollten Konzentration und Laufrate niedrig gehalten und das Mobilisationsregime explizit auf die motorische Restschwäche abgestimmt werden, einschließlich strukturierter Mobilisation nur unter pflegerischer Aufsicht und mit Gehhilfe.

#### FICB-Katheter.

In der Pilotarbeit von Mouzopoulos et al. [27] zur Delirprophylaxe konnte ein kontinuierlicher FICB bei intermediärer Risikogruppe die Delirinzidenz signifikant senken. Eine retrospektive Kohortenstudie an einem spezialisierten Zentrum für Hüftchirurgie zeigte, dass ein kontinuierlicher FICB im Vergleich zu konventioneller Analgesie ohne regionalen Block mit reduziertem Opioidverbrauch, niedrigerer Delirinzidenz und kürzerer Liegedauer assoziiert war [28]. Auch der FICB-Katheter geht mit einer relevanten, wenn auch in der Regel etwas geringeren Quadrizepsschwäche einher als der cFNB, was bei der Mobilisationsplanung berücksichtigt werden muss.

#### PENG-Katheter.

Theoretisch und praktisch attraktiv ist die Kombination einer effektiven Analgesie mit weitgehendem Erhalt der motorischen Funktion durch den kontinuierlichen PENG-Block. Erste Beobachtungsstudien zeigen Praktikabilität und gute Schmerzkontrolle [25]. Größere randomisierte Vergleichsdaten gegen FICB- oder Femoraliskatheter stehen noch aus, doch ist diese Technik aus pathophysiologischer Sicht das wahrscheinlich beste Konzept für unsere Patientengruppe und wird in spezialisierten Zentren zunehmend bevorzugt.

Aus pragmatischer Sicht eignen sich Schmerzkatheter besonders bei vorhersehbar längerer Wartezeit auf die Operation, bei multimorbiden Patienten mit hoher Empfindlichkeit gegenüber Opioiden sowie bei Patienten mit ausgeprägt schmerzhafter Frakturmorphologie. Die Logistik (sterile Anlage, klare Verantwortung, regelmäßige Visite, definierte Liegedauer in der Regel 24 bis 48 h, dokumentierter motorischer Status vor jeder Mobilisation) muss vor Implementierung interdisziplinär festgelegt werden. Bei der Wahl der Technik ist die Balance zwischen analgetischer Wirksamkeit und motorischer Restkapazität entscheidend: Wo immer der frühe Mobilisationspfad der dominante Outcome ist, ist der PENG-Katheter dem klassischen Femoraliskatheter vorzuziehen.

## Postoperatives Delir: Ein vermeidbares Schlüsselereignis

Das postoperative Delir ist die häufigste und zugleich konsequenzenreichste Komplikation nach hüftgelenknaher Fraktur. Berichtete Inzidenzen variieren je nach Erfassungsinstrument und Population zwischen 16 und 70 % [6, 19]. Das Delir ist ein eigenständiger Prädiktor erhöhter Letalität, schlechterer funktioneller Erholung und verlängerter Liegedauer [6].

Die klassischen Risikofaktoren (höheres Lebensalter, kognitive Vorschädigung, Polypharmazie, Komorbiditätslast, sensorische Defizite) sind dem Behandlungsteam in der Regel bekannt. Modifizierbar sind hingegen die perioperativen Trigger: Ungenügende Schmerzkontrolle, Opioidexposition, prolongierte Nüchternheit, Volumendepletion, Schlafentzug, Immobilisation, sensorische Deprivation und vermeidbare Polypharmazie (Benzodiazepine, anticholinerge Medikamente; [6, 19, 20]).

Die Evidenz spricht klar gegen monothematische Interventionen und für multimodale Präventionsbündel. Eine Metaanalyse zum FICB bei alten Patienten ohne präoperative kognitive Beeinträchtigung zeigte eine signifikante Reduktion der Delirinzidenz (Odds Ratio 0,46; [22]). Eine Beobachtungsstudie zur Implementierung eines optimierten klinischen Pfades, der u. a. einen FICB enthielt, dokumentierte eine signifikante Senkung der Delirrate sowie weiterer Komplikationen [29]. Auch der Ersatz oder die Reduktion postoperativer Opioide durch intravenöses Paracetamol als Teil eines multimodalen Schemas ging in einer Kohortenstudie mit weniger Delirepisoden einher [20]. Ein randomisierter Pilot zur perioperativen antiinflammatorischen Bündelstrategie bestätigt das Konzept, dass die Reduktion der systemischen Inflammation einen mediativen Effekt auf das Delirrisiko hat [30].

Aus diesen Daten lässt sich ein praxistaugliches Antidelirbündel formulieren, das mit den drei Themen dieser Arbeit unmittelbar verzahnt ist: keine vermeidbar verlängerte Nüchternheit, eine euvoläme Ausgangslage, eine multimodale Schmerztherapie mit früher peripherer Blockade (gegebenenfalls mit Katheter), Verzicht auf Benzodiazepine und Vermeidung anticholinerger Substanzen, ergänzt durch nicht-pharmakologische Maßnahmen (Reorientierung, Hör- und Sehhilfen, Tag-Nacht-Rhythmus, Mobilisation am Operationstag, Familienpräsenz).

## Synthese: Das Bündel als Wirkprinzip

Die diskutierten Maßnahmen entfalten ihre Wirkung weniger einzeln als im Verbund. Genau dies ist das konzeptionelle Herz des Enhanced-recovery-Gedankens, der für die elektive Hüftgelenkchirurgie bereits etabliert ist und sich zunehmend auch für die Notfallversorgung der Hüftfraktur durchsetzt [17, 18]. Eine systematische Übersicht und Metaanalyse von Lee et al. [18] aus dem Jahr 2025 zeigt für ERAS-Bündel in der orthopädischen Hüftchirurgie kürzere Verweildauern, niedrigere Komplikationsraten und eine bessere funktionelle Erholung. Eine Propensity-Score-gematchte Analyse bei intertrochantärer Fraktur durch Zhu et al. [31] unterstützt diese Befunde.

Die Tab. 1 fasst ein praxistaugliches perioperatives Bündel zusammen, das die hier diskutierten Stellschrauben integriert. Tab. 2 vergleicht die wichtigsten peripheren Nervenblockaden im Hinblick auf klinisch relevante Aspekte.

**Tab. 1:** Phasenspezifisches perioperatives Bündel bei trochantärer Femurfraktur

Phase	Empfohlene Maßnahmen
*Notfallaufnahme*	Frühe strukturierte Schmerzerfassung (NRS in Ruhe und Bewegung); Paracetamol intravenös als Basismedikation; restriktive niedrigdosierte Opioidgabe; Einleitung „sip till send“ mit Bettkennzeichnung; rasche orale Rehydrierung, gegebenenfalls 500–1000 ml balancierter Vollelektrolytlösung; ultraschallgeführter FICB oder PENG-Block durch geschulten Notfall‑/Anästhesiearzt
*Station präoperativ*	Fortsetzung „sip till send“ bis Theaterabruf; tägliche Bilanzierung und Gewicht; Vermeidung von Benzodiazepinen und anticholinergen Substanzen; Reorientierung und Familieneinbezug; Operation idealerweise innerhalb 24, spätestens 48 h; bei längerer Wartezeit kontinuierlicher FICB- oder PENG-Katheter erwägen
*Operation*	Spinalanästhesie oder Allgemeinanästhesie nach Patientenpräferenz und Komorbidität; zielorientierte Volumentherapie mit balancierten Kristalloiden, Vermeidung liberaler Volumengabe (> 1500 ml); restriktive Opioidexposition; Wärmemanagement; Tranexamsäure nach institutionellem Protokoll
*Postoperativ*	Frühe orale Flüssigkeits- und Nahrungsaufnahme; Mobilisation am Operationstag; multimodale Analgesie mit Paracetamol als Basis und Opioiden bei Bedarf in niedriger Dosierung; Fortsetzung des regionalen Katheters für 24 bis 48 h; tägliches Delirscreening (z. B. CAM oder 4AT); Frührehabilitation und orthogeriatrische Co-Visite

*FICB* Fascia-iliaca-compartment-Block, *PENG-Block* Pericapsular-nerve-group-Block

**Tab. 2:** Vergleich peripherer Nervenblockaden bei trochantärer Femurfraktur

Aspekt	Femoralisblock (FNB)	FICB infrainguinal	FICB suprainguinal	PENG-Block
*Erfasste Nerven*	N. femoralis (selektiv)	N. femoralis, N. cutaneus femoris lateralis (variabel)	N. femoralis, N. cutaneus femoris lateralis, N. obturatorius (verbessert)	Artikuläre Äste von N. femoralis, N. obturatorius, N. obturatorius accessorius
*Motorische Schwäche Quadrizeps*	Ausgeprägt; bei Katheter 70–80 % MVIC-Reduktion	Häufig	Häufig	In der Regel erhalten
*Sturzrisiko bei früher Mobilisation*	Erhöht, insbesondere unter kontinuierlicher Infusion	Erhöht	Erhöht	Niedrig
*Lagerung für Spinalanästhesie*	Erleichtert	Erleichtert	Deutlich erleichtert	Deutlich erleichtert
*Eignung Notfallaufnahme*	Sehr gut, technisch einfach	Sehr gut	Sehr gut	Gut, etwas anspruchsvollere Sonoanatomie
*Eignung Schmerzkatheter*	Etabliert; Quadrizepsschwäche limitierend	Etabliert	Etabliert, gut beschrieben	Zunehmend, jüngere Evidenz, motorisch sparend
*Studienlage*	Sehr umfangreich; gegen FICB weitgehend äquivalent	Umfangreich (Cochrane, multiple RCT)	Umfangreich, teils überlegen gegenüber infrainguinal	Wachsend; mehrere RCT und aktuelle Metaanalyse

*FICB* Fascia-iliaca-compartment-Block, *PENG-Block* Pericapsular-nerve-group-Block, *FNB* Femoralisblock, *RCT* „randomised controlled trial“

## Stärken und Limitationen der Evidenz

### Stärken.

Die Evidenz für die hier diskutierten Maßnahmen ist bemerkenswert konsistent. Mehrere unabhängige Metaanalysen belegen die opioidsparende und delirreduzierende Wirkung peripherer Nervenblockaden [21, 22, 25]. Die Sicherheit verkürzter Nüchternheitszeiten ist sowohl pathophysiologisch plausibel als auch durch Beobachtungs- und Implementierungsstudien gestützt [9, 11, 13]. Die Daten zur Volumentherapie zeigen einen plausiblen Dosis-Wirkungs-Zusammenhang in die ungünstige Richtung bei liberaler Gabe [16].

### Limitationen.

Die Mehrzahl der zitierten Studien fasst Schenkelhals- und trochantäre Frakturen als hüftnahe Frakturen zusammen, die spezifische Subgruppe der trochantären Fraktur wird selten getrennt ausgewertet. Die Evidenz zu „sip till send“ stützt sich überwiegend auf Qualitätsverbesserungsdaten aus dem britischen und australischen Raum mit großem Zentrumsbias; randomisierte Vergleiche zur klassischen Karenz fehlen aus methodischen und ethischen Gründen weitgehend. Die ERAS-Bündel sind heterogen, was die Generalisierbarkeit einzelner Ergebnisse erschwert. Schließlich liegen für die kontinuierlichen PENG-Katheter bisher überwiegend kleine Serien und einzelne randomisierte Studien vor; größere multizentrische Daten stehen aus.

## Schlussbetrachtung

Die trochantäre Femurfraktur ist ein Eingriff, dessen Ergebnis nicht erst im Operationssaal entschieden wird. Drei Stellschrauben, die in der Diskussion um Implantatwahl, Augmentation und Antikoagulation regelmäßig in den Hintergrund geraten, prägen den Verlauf erheblich: die präoperative Nüchternheit, die Volumentherapie und das Schmerzmanagement.

Klare Flüssigkeiten bis zur Abrufung in den Saal sind sicher und vorteilhaft. Eine zielorientierte Volumentherapie mit balancierten Kristalloiden vermeidet sowohl Hypovolämie als auch die mit liberaler Gabe assoziierten Komplikationen. Periphere Nervenblockaden, insbesondere FICB suprainguinal und PENG-Block, gehören in die Standardversorgung; bei längerer Wartezeit oder besonders schmerzhafter Frakturmorphologie ist ein kontinuierlicher Katheter eine wertvolle Option. Die konsequente Bündelung dieser Maßnahmen reduziert das Auftreten des postoperativen Delirs und ist mit kürzerer Verweildauer und besserer funktioneller Erholung assoziiert.

Die Aufgabe für die orthopädisch-unfallchirurgische Klinikleitung besteht weniger im Studium neuer Implantate als in der konsequenten interdisziplinären Verankerung dieser Selbstverständlichkeiten in einem schriftlich konsentierten perioperativen Pfad, getragen von Anästhesie, Orthopädie/Unfallchirurgie, Pflege, Geriatrie und Physiotherapie.

## Fazit für die Praxis

- „Sip till send“ (klare Flüssigkeit bis zum Saalabruf) ist sicher und sollte bei trochantärer Femurfraktur die Standardvorgabe sein, abweichend nur nach individueller anästhesiologischer Risikoabwägung.
- Eine liberale intraoperative Volumengabe (> 1500 ml) ist mit erhöhter Komplikationsrate assoziiert. Empfohlen wird eine zielorientierte Volumentherapie mit balancierten Kristalloiden.
- Periphere Nervenblockaden (FICB [Fascia-iliaca-Compartment-Block] suprainguinal, PENG-Block [Pericapsular-nerve-group-Block]) reduzieren Schmerz und Opioidbedarf und senken die Inzidenz des postoperativen Delirs.
- Bei längerer präoperativer Wartezeit oder anhaltend hohem Analgetikabedarf ist ein kontinuierlicher Schmerzkatheter (FICB- oder PENG-Katheter) eine attraktive Option.
- Das postoperative Delir ist ein modifizierbares Schlüsselereignis. Ein integriertes Bündel aus kurzer Nüchternheit, euvoläme Ausgangslage, multimodaler Analgesie und nicht-pharmakologischen Maßnahmen ist die wirksamste Prävention.

## Data Availability

Alle dieser Arbeit zugrunde liegenden Daten sind in diesem Artikel enthalten.
